# Improved production of Humira antibody in the genetically engineered *Escherichia coli* SHuffle, by co-expression of human PDI-GPx7 fusions

**DOI:** 10.1007/s00253-020-10920-5

**Published:** 2020-09-30

**Authors:** Marine Lénon, Na Ke, Cecily Szady, Hassan Sakhtah, Guoping Ren, Bruno Manta, Bryce Causey, Mehmet Berkmen

**Affiliations:** 1 New England Biolabs, 240 County Road, Ipswich, MA 01938 USA; 2 Present Address: Department of Microbiology, Stress Adaptation and Metabolism in Enterobacteria Unit, UMR CNRS 2001, Institut Pasteur, 25-28 Rue du Dr Roux, 75015 Paris, France; 3 Present Address: Boston Institute of Biotechnology, LLC., Upstream Process Development, 225 Turnpike Road, Southborough, MA 01772 USA; 4 Present Address: Facultad de Medicina, Departamento de Bioquímica and Centro de Investigaciones Biomédicas, Universidad de la República, CP 11800 Montevideo, Uruguay

**Keywords:** SHuffle, GPx7, PDI, Redox engineering, Humira production, Disulfide bond, *E. coli*

## Abstract

**Abstract:**

Microbial production of antibodies offers the promise of cheap, fast, and efficient production of antibodies at an industrial scale. Limiting this capacity in prokaryotes is the absence of the post-translational machinery, present in dedicated antibody producing eukaryotic cell lines, such as B cells. There has been few and limited success in producing full-length, correctly folded, and assembled IgG in the cytoplasm of prokaryotic cell lines. One such success was achieved by utilizing the genetically engineered *Escherichia coli* strain SHuffle with an oxidative cytoplasm. Due to the genetic disruption of reductive pathways, SHuffle cells are under constant oxidative stress, including increased levels of hydrogen peroxide (H_2_O_2_). The oxidizing capacity of H_2_O_2_ was linked to improved disulfide bond formation, by expressing a fusion of two endoplasmic reticulum-resident proteins, the thiol peroxidase GPx7 and the protein disulfide isomerase, PDI. In concert, these proteins mediate disulfide transfer from H_2_O_2_ to target proteins via PDI-Gpx7 fusions. The potential of this new strain was tested with Humira, a blockbuster antibody usually produced in eukaryotic cells. Expression results demonstrate that the new engineered SHuffle strain (SHuffle2) could produce Humira IgG four-fold better than the parental strain, both in shake-flask and in high-density fermentation. These preliminary studies guide the field in genetically engineering eukaryotic redox pathways in prokaryotes for the production of complex macromolecules.

**Key points:**

*• A eukaryotic redox pathway was engineered into the E. coli strain SHuffle in order to improve the yield of the blockbuster antibody Humira.*

*• The best peroxidase-PDI fusion was selected using bioinformatics and in vivo studies.*

*• Improved yields of Humira were demonstrated at shake-flask and high-density fermenters.*

**Electronic supplementary material:**

The online version of this article (10.1007/s00253-020-10920-5) contains supplementary material, which is available to authorized users.

## Introduction

Antibody-based technologies is an expanding field in biotech and pharmaceutical industries with a myriad of applied tools in biological research. Microbial production of therapeutic and engineering of diagnostic antibody derivatives offers great potential for cheap, fast production of novel antibody designs. Limiting this potential is the correct folding and assembly of the tetrameric antibody (IgG). The production of an IgG macromolecule demands the assembly of two light and two heavy chains to be stoichiometrically associated, along with the requirement for post-translational modifications of proline isomerization and glycosylation and for 8 intra and 2 inter-chain disulfide bonds (Feige et al. 2009). Consequently, the production of IgG is not a trivial problem especially in the context of a prokaryotic expression system.

In the model prokaryotic expression organism *Escherichia coli*, expression of antibodies and derivatives has been limited to the periplasmic compartment (Simmons et al. 2002) mainly due to its native disulfide bond–forming machinery (Manta et al. 2019). Yet, periplasmic expression requires extensive optimization of conditions (Baumgarten et al. 2018) to avoid secretion blockage (Schlegel et al. 2012) and optimization of targeting signal peptides (Karyolaimos et al. 2019; Mirzadeh et al. 2020). More importantly, the periplasm lacks ATP (Alvarez et al. 2017) and thus is devoid of ATP-dependent chaperone systems, making the cytoplasm the preferred compartment for protein expression.

The cytoplasm of *E. coli* contains two disulfide bond–reducing pathways, an unsuitable compartment for the production of disulfide-bonded proteins. The problem of the reductive compartment of the cytoplasm was circumvented with the redox engineering of the cytoplasmic redox pathway of *E. coli*, resulting in the protein expression strain, SHuffle (Lobstein et al. 2012) or by co-expressing a eukaryotic oxidase pathway (Gaciarz et al. 2017). SHuffle cells have diminished disulfide bond reducing power due to the deletion of thioredoxin reductase (*trxB*) and glutathione reductase (*gor*) along with a suppressor mutation in the peroxidase AhpC (Ritz et al. 2001).

Redox engineered *E. coli* SHuffle cells have been previously demonstrated to be an attractive platform for the expression of various antibody formats, such as full-length IgG (Reddy et al. 2018; Robinson et al. 2015), Fab’ fragments (Abe et al. 2014; Mori et al. 2018; Yusakul et al. 2018), scFv chains (Ahmadzadeh et al. 2020; Liu et al. 2019; Vermeulen et al. 2018) and VHH domains (Eliseev et al. 2018; Ta et al. 2015; Zarschler et al. 2013). Although SHuffle cells lack the eukaryotic glycosylation machinery, by engineering mutations in the Fc region of antibodies, the requirement for glycosylation for efficient binding of the IgG to its cognate receptor was bypassed (Robinson et al. 2015). Furthermore, unlike mammalian or yeast cells, expression of antibodies in SHuffle cells permits the efficient labelling of antibodies with heavy isotopes for structural studies (Reddy et al. 2018). A microbial platform for the design, selection, and production of antibodies in a rapid manner, especially in response to an emerging infection is therefore essential. Not surprisingly, many new methods and strains have been developed to increase the microbial capacity of recombinant antibody production (Gupta and Shukla 2017; Spadiut et al. 2014; Zhang et al. 2020). In this study, the genetic tools available to *E. coli* were utilized to engineer synthetic eukaryotic redox pathway and evaluate its effect on the production of the most profitable and widely used therapeutic antibody Humira (adalimumab) against human tumor necrosis factor alpha (TNFα), used as a blocker to treat rheumatoid arthritis (Scheinfeld 2003).

SHuffle cells are under constant oxidative stress, presumably due to loss of peroxidase activity of AhpC, resulting in the accumulation of hydrogen peroxide (H_2_O_2_) (Reuter et al. 2019). The accumulation of H_2_O_2_ is known to cause oxidative stress (Hong et al. 2019) and may not only damage the proteome of SHuffle cells but also perturb recombinant protein expression. This insight allowed us to postulate linking the oxidizing capacity of H_2_O_2_ to disulfide bond formation using the endoplasmic resident eukaryotic glutathione peroxidase-7 (GPx7) and the enzyme responsible for oxidative folding, the Protein Disulfide bond Isomerase (PDI).

The peroxidase superfamily present in all domains of life can be divided into 8 subgroups (Toppo et al. 2008). GPx7 is an endoplasmic reticulum (ER)-resident peroxidase that contributes to oxidative protein folding by reducing H_2_O_2_ and donating its disulfide bond to PDI (Wang et al. 2014) (Nguyen et al. 2011). Oxidized PDI participates in oxidative folding of proteins both in vivo and in vitro (Wang et al. 2014).

Engineering of a eukaryotic PDI-GPx7 coupled redox pathway is therefore an attractive option, as the components may not need to interact with a prokaryotic system. In this study, an attempt was made to genetically engineer a eukaryotic redox pathway that naturally resides in ER, to express and function in the cytoplasm of a previously engineered prokaryotic cell, SHuffle. Accumulated H_2_O_2_ pools were coupled to disulfide bond formation by the co-expression of PDI-GPx7 fusions. The feasibility of this redox-coupled system was demonstrated on the most profitable therapeutic protein, Humira IgG. Co-expression of human PDI-GPx7 fusion improved the correct assembly and yield of Humira IgG in high-density fermentations by several folds.

## Materials and methods

### *E. coli* strains and plasmids

Bacterial strains and plasmids used in this work are described in Table 1 and were constructed using a standard molecular and genetic technique (Sambrook et al. 1989). NEB 10-beta competent *E. coli* (New England Biolabs, cat. No. C3019) was used for plasmid cloning procedures and transformed by heat shock transformation, following the manufacturer’s instructions. Transformed cells were then selected on LB agar plates containing 30 μg/mL chloramphenicol or 200 μg/mL ampicillin where appropriate. SHuffle B T7 express *E. coli* cells (New England Biolabs, cat. No. C3029) were used as hosts for protein expression and purification studies.

**Table 1:** Bacterial strains and plasmids utilized in this study

**Strains**	**Relevant genotype**		**Source**
SHuffle express T7 (C3029)	*E. coli BL21 fhuA2 lacZ::T7 gene1 [lon] ompT ahpC gal λatt::pNEB3-r1-cDsbC* (Spec^R^, *lacI*^*q*^) *ΔtrxB sulA11 R(mcr-73::miniTn10--*Tet^S^*)2 [dcm] R(zgb-210::Tn10 --*Tet^S^*) endA1 Δgor ∆(mcrC-mrr)114::IS10*		NEB cat# C3029
MB2797	C3029 + pBAD34 - KatG-FLAG		This study
MB4638	C3029 + pACYC Duet		This study
MB6226	C3029 + pACYC Duet - PDI-hsa Gpx7-Flag (SHuffle2)		This study
MB6227	C3029 + pACYC Duet - PDI-mmu Gpx7-Flag		This study
MB6228	C3029 + pACYC Duet - PDI-dre Gpx7-Flag		This study
MB6229	C3029 + pACYC Duet - PDI-lak Gpx7-Flag		This study
MB6230	C3029 + pACYC Duet - PDI-aqu Gpx7-Flag		This study
MB6231	C3029 + pACYC Duet - PDI-gga Gpx7-Flag		This study
MB6232	C3029 + pACYC Duet - PDI-pxb Gpx7-Flag		This study
MB6233	C3029 + pACYC Duet - cPDI-Gpx7-Flag		This study
MB6165	C3029 + pACYC Duet	+ pET23b	This study
MB4135	C3029	+ pHumira	This study
MB6209	C3029 + pACYC Duet	+ pHumira	This study
MB6201	C3029 + pACYC Duet - PDI-hsa Gpx7	+ pHumira (SHuffle2)	This study
MB6202	C3029 + pACYC Duet - PDI-mmu Gpx7	+ pHumira	This study
MB6203	C3029 + pACYC Duet - PDI-dre Gpx7	+ pHumira	This study
MB6204	C3029 + pACYC Duet - PDI-lak Gpx7	+ pHumira	This study
MB6205	C3029 + pACYC Duet - PDI-aqu Gpx7	+ pHumira	This study
MB6206	C3029 + pACYC Duet - PDI-gga Gpx7	+ pHumira	This study
MB6207	C3029 + pACYC Duet - PDI-pxb Gpx7	+ pHumira	This study
MB6208	C3029 + pACYC Duet - cPDI-Gpx7	+ pHumira	This study
MB6210	C3029 + pACYC Duet - Gpx7-hsa-Flag	+ pHumira	This study
**Plasmids**	**Features**		**Source**
pHumira	Humira antibody cloned into pETDuet NdeI/XhoI site by Gibson Assembly of PCR product of LC and HC, under the regulation of T7 promoter, pBR322 origin, AmpR.		(Leith et al. 2019)
pACYC Duet	Vector encodes two multiple cloning sites (MCS) under the control of T7 promoter, lac operator and ribosome binding site. pACYC origin, CamR.		Novagen cat# 71146–3
pACYC Duet-PDI-lak GPx7-FLAG	*E. coli* codon optimized PDI-GPx7 fusion from *Lingula anatina* (PDI Accession Number XP_013416130.1 and GPx7 Accession Number XP_013409653.1) was synthesized and cloned into pACYC Duet (*Nco*I/*Sal*I). 3’ Flag tag was inserted using the primers NK1012-FLAG-r and NK1016-lak-f. pACYC origin, CamR.		This study
pACYC Duet-PDI-pxb GPx7-FLAG	*E. coli* codon optimized PDI-GPx7 fusion from *Pyrus x bretschneideri* (Chinese white pear, PDI Accession Number XP_009335086.1 and GPx7 Accession Number XP_009359105.1) was synthesized and cloned into pACYC Duet (*Nco*I/*Sal*I). 3’ Flag tag was inserted using the primers NK1012-FLAG-r and NK1014-pxb-f. pACYC origin, CamR.		This study
pACYC Duet-PDI-aqu GPx7-FLAG	*E. coli* codon optimized PDI-GPx7 fusion from *Amphimedon queenslandica* (Sponge PDI Accession Number XP_003382810.1 and GPx7 Accession Number XP_003388439.1) was synthesized and cloned into pACYC Duet (*Nco*I/*Sal*I). 3’ Flag tag was inserted using the primers NK1012-FLAG-r and NK1015-aqu-f. pACYC origin, CamR.		This study
pACYC Duet-PDI-dre GPx7-FLAG	*E. coli* codon optimized PDI-GPx7 fusion from *Danio rerio* (Zebrafish PDI Accession Number NP_998529.3 and GPx7 Accession Number NP_001018337.1) was synthesized and cloned into pACYC Duet (*Nco*I/*Sal*I). 3’ Flag tag was inserted using the primers NK1012-FLAG-r and NK1017-dre-f. pACYC origin, CamR.		This study
pACYC Duet-PDI-gga GPx7-FLAG	*E. coli* codon optimized PDI-GPx7 fusion from *Gallus gallus* (Chicken PDI Accession Number NP_001185639.2 and GPx7 Accession Number NP_001156717.1) was synthesized and cloned into pACYC Duet (*Nco*I/*Sal*I). 3’ Flag tag was inserted using the primers NK1012-FLAG-r and NK1018-gga-f. pACYC origin, CamR.		This study
pACYC Duet-PDI-hsa GPx7-FLAG	*E. coli* codon optimized PDI-GPx7 fusion from *Homo sapiens* (Human PDI Accession Number NP_000909.2 and GPx7 Accession Number NP_001139309.1) was synthesized and cloned into pACYC Duet (*Nco*I/*Sal*I). 3’ Flag tag was inserted using the primers NK1012-FLAG-r and NK1020-hsa-f. pACYC origin, CamR.		This study
pACYC Duet-PDI-mmu GPx7-FLAG	*E. coli* codon optimized PDI-GPx7 fusion from *Mus musculus* (Mouse PDI Accession Number NP_035162.1 and GPx7 Accession Number NP_077160.1) was synthesized and cloned into pACYC Duet (*Nco*I/*Sal*I). 3’ Flag tag was inserted using the primers NK1012-FLAG-r and NK1019-mmu-f. pACYC origin, CamR.		This study
pACYC Duet-hsa-GPx7- FLAG	*E. coli* codon optimized GPx7 from *Homo sapiens* (Human GPx7 Accession Number NP_001139309.1) was synthesized and cloned into pACYC Duet (*Nco*I/*Hind*III) using the primers 5′-nsGpx7 and 3′-nsGpx7. pACYC origin, CamR.		This study

Low copy number pACYC Duet plasmids (Novagen cat. No. 71147) with a strong T7 promotor were used to express GPx7-PDI fusion genes (Table 1). The fusion constructs were designed in silico with a 17 amino acid linked in between PDI and GPx7 and synthesized by Genescript (www.genscript.com) with codons optimized for *E. coli* expression in pACYC Duet plasmid between *Nco*I and *Sal*I restriction sites. A FLAGX3 tag was introduced in 3′ end by PCR using the primers listed in Supplementary Table S1 and the description of the use of primers in constructing plasmids is listed in Supplementary Table S2.

### Shake-flask culture growth conditions

Overnight cultures of 5 mL Rich media (10 g/L soy peptone, 5 g/L yeast extract, 5 g/L NaCl, NaOH to pH 7.2) with the appropriate antibiotics were used to inoculate 1/100th *v*/v 25 mL shake-flask cultures and grown at 30 °C for 3 h until OD_600_ reached 0.8. The cultures were then induced with 500 μM isopropyl-β-D-thiogalactopyranoside (IPTG) and grown at 16 °C overnight or for 5 h at 30 °C with shaking at 220 rpm. Cells were harvested by centrifugation and frozen at − 20 °C. Cells densities were then standardized to the same ratio OD_600_ value using lysis buffer (1× PBS, 5% glycerol, 1 mM EDTA) and consequently lysed by sonication. The total fraction (*T*) was then centrifuged for 15 min at 20,000×*g* 4 °C. Proteins in the soluble fraction (*S*) were quantified with BCA reagent (BCA Protein Assay kit, Pierce, cat. No. 23225). The expression and activity of the oxidative substrate were then analyzed by western blot and/or ELISA assays, where appropriate.

### High-density fermentation growth conditions

Seed cultures were inoculated by adding a single colony from the streaked plates into 250 mL Erlenmeyer flasks containing 125 mL of selective LB liquid medium. The seed cultures were grown at 30 °C, shaking at 275 rpm for 14 h, and then immediately used to inoculate DASGIP® BioBlock fermenters containing 1 L complex medium (1.2% soytone, 2.4% yeast extract, 4 mM potassium hydroxide, 2% glycerol, 25 mM dibasic potassium phosphate, 0.05% antifoam 204, 1× DeLissa Trace metals, and selective antibiotics). The fermenters were controlled by a computer running DASware software (Eppendorf, Hauppauge, NY). The pH was maintained at 7.0 using 28% ammonium hydroxide and 10% phosphoric acid. The cultures were grown at 30 °C and agitated at an initial speed of 500 rpm. When the dissolved oxygen reached 30% air saturation, an agitation/gas flow/oxygen enrichment cascade was used to maintain this level of oxygenation. After 9 to 10 h of growth, IPTG was added to a final concentration of 0.4 mM in the fermenter. One milliliter of culture was sampled throughout the experiments and used to monitor metabolite production/nutrient consumption and recombinant protein production. After recording the density of the culture, the samples were centrifuged at 13,000 rpm for 75 s, and the cell pellet was collected and stored at − 20 °C. Cells were resuspended into lysis buffer at OD_600_ = 50. 1 mL of the resuspended cells was sonicated at 40% maximum amplitude for 2 min at 2 s on and 4 s off setting for three rounds. Insoluble matter was removed by 15 min and 13,000 rpm centrifugation and the soluble supernatant fractions were collected.

### PDI and GPx7 bioinformatic analysis

Mouse GPx7 sequence (NP_077160.1) without the ER-retention signal was used as bait to search NCBI nr database restricted to eukaryotes via DELTA-BLAST (Boratyn et al. 2013). Retrieved sequences were manually curated for domain topology, ER-retention signal, and length using SMART (http://smart.embl-heidelberg.de, (Letunic and Bork 2011) and Signal P (http://www.cbs.dtu.dk/services/SignalP, (Petersen et al. 2011). Sequences were aligned with Clustal Omega (http://www.ebi.ac.uk/Tools/msa/clustalo, (Sievers et al. 2011) and trees made with same program, using default parameter (Blackshields et al. 2010). Dendrograms were plotted with iToL (http://itol.embl.de, (Letunic and Bork 2011)) and manually edited for presentation. To clone the respective PDI homologs from each selected organism, the closest homolog to human protein disulfide isomerase precursor (PDI, NP_000909) were chosen and cloned 5′ to its respective GPx7. A synthetic consensus sequence of GPx7 and PDI was designed by retaining the conserved residues of the aligned homologs based on previously described methods (Sternke et al. 2019).

### Redox state analysis

The in vivo redox state of the fusion proteins was determined by trapping free thiol groups with 4-acetamido-4′-maleimidylstilbene-2,2′-disulfonic acid (AMS, Life Technologies, cat. No. A-485) following published protocols (Ke and Berkmen 2014). Briefly, 1 mL of induced culture at OD_600_ 0.5 was transferred to 1.5 mL tubes containing 0.2 mL of 100% Trichloroacetic Acid (TCA, Fisher Scientific, cat. No. A322). Samples were mixed thoroughly by vortexing and incubated on ice for at least 20 min. Precipitated proteins were centrifuged 15 min at 16,000×*g* at 4 °C and 0.6 mL cold acetone was added to the pellet. Samples were vortexed briefly and incubated for 20 min on ice. After the wash step, proteins were centrifuged 15 min at 16,000×*g* at room temperature and the pellet was air dried for 15 min. Three experiments were prepared for each sample. As a positive control for AMS alkylation, precipitated samples were dissolved in 0.2 mL of 100 mM Tris-Cl pH 6.8 with 0.1 mM DTT and 1% SDS. After complete resuspension of the protein precipitate in 0.9 mL of 100 mM Tris-Cl, pH 8.0, samples were added to 0.2 mL 100% TCA to repeat the protein precipitation step. DTT was omitted in the test sample. Precipitated proteins from the positive control and test sample were resuspended in 80 μL of 100 mM Tris-Cl, pH 6.8 containing 10 mM AMS and 1% SDS. A negative control was included omitting AMS in the resuspension buffer. Proteins were completely dissolved by mixing tubes for 20 min at room temperature. The AMS alkylation reaction was then performed at 37 °C for 40 min. The oxidative state of the proteins was analyzed by immunoblotting using the primary anti-FLAG Tag (9A3) mouse mAb (Cell Signaling Technology, cat. No. 8146) and the secondary DyLight™ IgG (Cell Signaling Technology, anti-mouse 800 conjugate cat. No. 5257).

### Western blot

Protein samples were diluted in 1X Loading Buffer (New England Biolabs, cat. No. B7709) with or without DTT and loaded on Novex™ 4–20% Tris-Glycine pre-cast gel (ThermoFisher Scientific, WedgeWell™ format). SDS-PAGE was performed for 1 h at 45 mA per gel and proteins were transferred on PVDF membrane (BioRad, cat. No. 170-4157) using semi-dry blotting bio-rad protocol (BioRad, Trans-Blot® Turbo™ Blotting System, cat. No. 170–4155) for 7 min at 2.5 A. Membranes were blocked with Odyssey Blocking Buffer (LI-COR Biosciences, cat. No. 927–40,000) for 1 h at room temperature with gentle shaking and washed 3 times with PBS, 0.05% Tween for 5 min, with shaking. The primary antibody (anti-Flag-Tag (9A3) mouse mAb (Cell Signaling Technology, cat. No. 8146) was diluted in Odyssey Blocking Buffer with 0.2% Tween and incubated with the membrane overnight at 4 °C. The next day, membranes were washed as before and incubated with corresponding secondary DyLight™ IgG (Cell Signaling Technology, anti-mouse 800 conjugate cat. No. 5257) diluted in Odyssey Blocking Buffer, 0.2% Tween, 0.02% SDS for 1 h with gentle shaking, protected from light. After a last wash step, membranes were scanned on an Odyssey Imaging System.

### IgG purification

IgG was induced in SHuffle2, cells were harvested and the cell pellet was resuspended into 1 mL lysis buffer (1XPBS with 5% glycerol and 1 mM EDTA) to OD 50. The 1 mL cell suspension was sonicated with mini tipped sonicator for 1 min (4 s on and 2 s off) and repeated three times. The sonicated cell suspension was spun at 13,000 rpm for 15 min. The supernatant containing the soluble fractionation was separated from the insoluble pellet and subjected to purification. IgG was purified from the soluble fraction by affinity using protein A magnetic beads (New England Biolabs, cat. No. S1425) following the “Antibody Purification” protocol as recommended in the Pierce™ Protein A Magnetic Beads manual (ThermoFisher, MAN0011856), using Thermo Scientific KingFisher Flex instrument (ThermoFisher, 5,400,640). The low pH of the eluate was neutralized by adding 5 μL of Neutralization Buffer (20% Tris base pH 9.5) and purified fraction was resolved by SDS-PAGE under non-reducing and reducing conditions. Proteins bands were visualized by staining with SimplyBlue™ SafeStain (ThermoFisher Scientific, Invitrogen™, cat. No. LC6065). And yields of purified IgG was obtained by BCA assay (ThermoFisher, cat 23,225).

### ELISA

The total amount of purified IgG was analyzed by sandwich ELISA by using the Adalimumab ELISA kit for the low-density expression (Eagle Biosciences, cat. No. IG-AA103) and the BioSim™ anti-Adalimumab ELISA kit for the high-density expression (BioVision, cat. No. E4388). IgG-containing samples were quantified with BCA reagent and an equivalent amount of total protein (typically 0.23–0.06 μg) was applied to the plate. The procedure was followed as noticed in the manual of the ELISA assay kit and the optical density was measured at 450 nm within 15 after pipetting the stop solution.

### Nucleotide sequences

Nucleotide sequences of the *E. coli* codon optimized PDI-GPx7 fusions can be accessed via GenBank using the accession codes: For human (MT764745), mouse (MT764747), chicken (MT764744), zebrafish (MT764750), sponge (MT764749), pear (MT764748) and mollusk (MT764746) PDI-GPx7 fusions.

## Results

### Engineered redox pathway of SHuffle

**Fig. 1:**
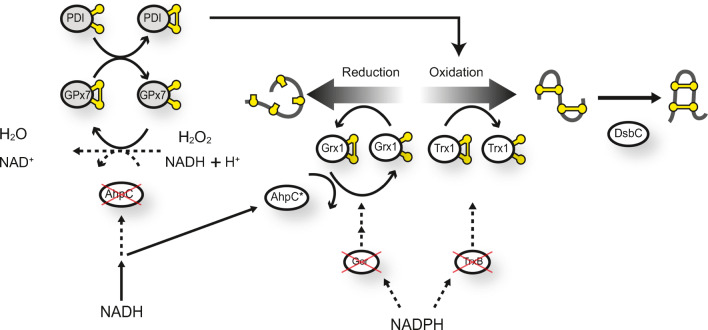
Schematic representation of the redox pathway in SHuffle2. Genetic deletions of *gor* and *trxB* genes disrupt the electron flow (dotted lines) to the glutathione (Grx) and thioredoxin (Trx) reductases, resulting in lethality. Suppressors are mapped to the mutant peroxidase AhpC*, which has lost its peroxidase activity and instead has gained the ability to reduce Grx1. Oxidized thioredoxin (Trx1) facilitates the oxidation of proteins, which are further isomerized by the cytoplasmic expression of disulfide bond isomerase, DsbC. The lack of peroxidase activity of AhpC* results in accumulation of H_2_O_2_. Expression of PDI-GPx7 fusions (here shown as separate proteins for clarity) results in the oxidation of GPx7 by H_2_O_2_, which in turn oxidizes PDI who can then participate oxidation

SHuffle cells have undergone genetic manipulations in order to optimize the formation of disulfide bonds in proteins expressed in the cytoplasm. The genetic manipulations can be summarized as disruption of the disulfide bond reductive pathways and selection for suppressors which permit the formation of disulfide bonds. Genes coding for thioredoxin reductase (*trxB*) and glutathione reductase (*gor*) were deleted. This double Δ*trxB,* Δ*gor* deletion is lethal as it abolishes the main reducing pathways responsible for maintaining the redox cycle of the cells. A suppressor of Δ*trxB*, Δ*gor* was selected to map to cytoplasmic alkyl hydroperoxide reductase C (AhpC) which has lost the function as a peroxidase and gained the ability to reduced glutathionylated glutaredoxin-1 (Yamamoto et al. 2008). To further enhance the fidelity of disulfide bond formation, a chromosomal copy of cytoplasmic DsbC (cDsbC) was inserted, resulting in the final Δ*trxB*, Δ*gor*, ΔahpC* + cDsbC strain, named SHuffle (Lobstein et al. 2012). The lack of peroxidase activity of AhpC* induces oxidative stress (Reuter et al. 2019), presumably due to the increased amounts of H_2_O_2_ (Fig. 1).

To engineer a redox pathway, originally localized in the ER of a eukaryote, we produced various PDI-GPx7 fusion proteins in the cytoplasm of a prokaryote. Fusion of PDI to GPx7 is assumed to increase the probability of a H_2_O_2_ → GPx7 → PDI → Humira redox relay pathway. This is mainly due to the transient and rapid cascades of oxidation by H_2_O_2_ (Wang et al. 2014). Consequently, by decreasing the proximity between GPx7 and PDI, H_2_O_2_ oxidized GPx7 would in turn oxidize PDI, recapitulating the PDI-GPx7 redox cycle in its native ER compartment (Roberto Sitia and Tobias Dick, personal communications) (Fig. 1).

### Bioinformatic selection of PDI-GPx7 fusion pairs

**Fig. 2:**
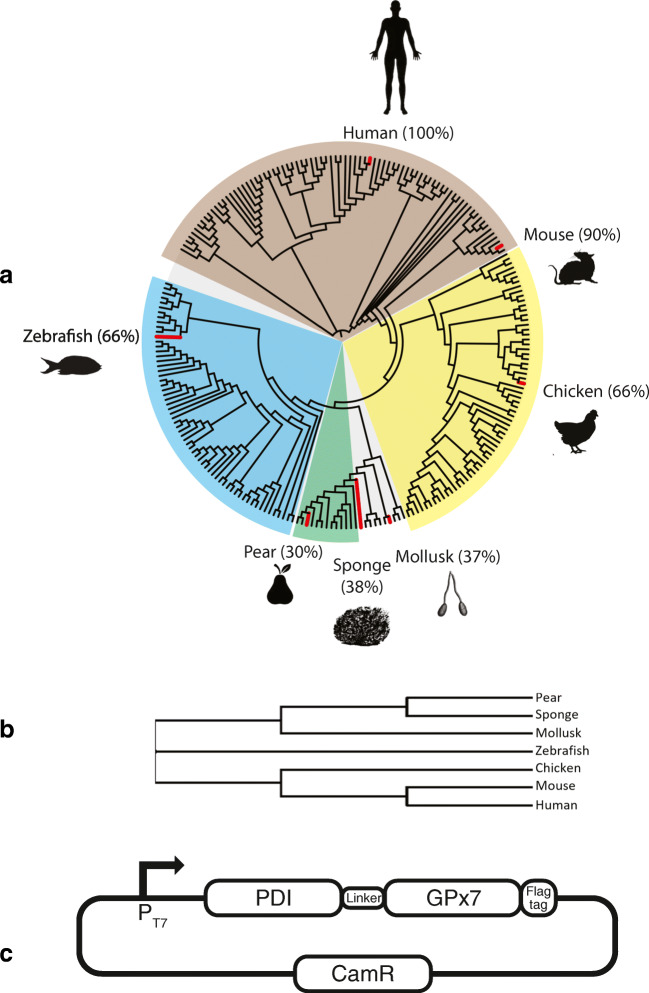
Selection of GPx7 homologs. **a** Phylogenetic distribution of GPx7 homologs and selected candidates are indicated. The percentage amino acid sequence identity to human GPx7 is shown in brackets. **b** Representative view of the phylogeny from the selected GPx7 homologs. The FastTree was made without branch length from the eight Gpx7 homolog sequences. **c** Schematic representation of the pACYC Duet vector expressing the PDI-GPx7 fusions. PDI and GPx7 peroxidases were fused by a poly-linker of 17 amino acids and cloned under the regulation of a T7 promoter with a C-terminal 3X Flag tag

The ability of GPx7 class of peroxidases to oxidize PDI has only been studied in the context of eukaryotic cells (Bosello-Travain et al. 2013; Laurindo et al. 2012; Maiorino et al. 2015). Due to minimal pre-existing data on *E. coli* expressed GPx7, little is known regarding which PDI-GPx7 fusion couple would express, fold, and be active when expressed in the cytoplasm of SHuffle. In order to maximize the chances of expressing the correct couple, a simple bioinformatic approach was used. Starting with mouse GPx7 (NP_077160.1), a BLAST search for homologs was performed. To construct a simple phylogenetic tree, ClustalW was used to construct a sequence alignment and the final tree was visualized using iTol (Fig. 2a). From this tree, seven homologs of GPx7 were chosen from separate phylogenetic nodes, spanning over the entire phylogenetic tree; Human (*Homo sapiens*), Mouse (*Mus musculus*), Chicken (*Gallus gallus*), Zebrafish (*Danio rerio*), Sponge (*Amphimedon queenslandica*), Pear (*Pyrus x bretschneideri*) and Mollusk (*Lingula anatina*) (Fig. 2b). A consensus sequence of GPx7 and PDI was also synthesized as described in materials and methods. Selected homologs all share (1) conserved active site cysteines, (2) N-terminal signal peptide, (3) C-terminal ER KDEL retention signal peptide, and (4) have conserved dimer interface sequence (Supplementary Fig. S1).

To construct the selected GPx7’s fused to their cognate PDI partner, human PDI (PDI, NP_000909) was used to BLAST within the genomes of the selected organisms and those with highest homology were chosen. PDI was chosen as the N-terminal fusion as simple structural analysis indicated that PDI-linker-GPx7 fusion is more favorable to allow the active sites of both PDI and GPx7 to potentially face each other. The final PDI-GPx7 couple was cloned as a fusion protein, using the 17 amino acid linker GSGSGSGSGSGSSGSGS (Foit et al. 2009), along with a C-terminal Flag tag and cloned into pACYC-Duet plasmid under the regulation of the strong T7 promoter (Fig. 2c).

### Expression of PDI-GPx7 fusion pairs

**Fig. 3:**
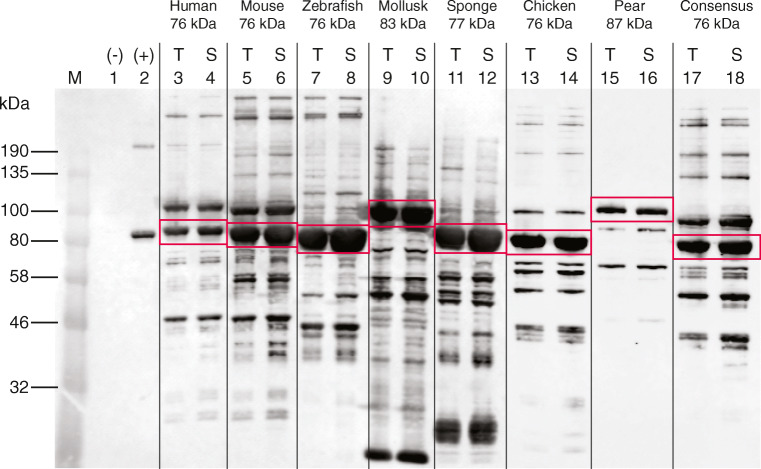
SHuffle expressed Gpx7-PDI fusions are soluble. SHuffle cells expressing various PDI-GPx7 fusions were lysed by sonication (total fraction, *T*) and the insoluble fraction was removed by centrifugation, separating the soluble supernatant fraction (*S*). Cells expressing empty vector was used as negative control (lane 1) and cells expressing flag-tagged KatG (83 kDa) was used as a positive control for the western blot (lane 2). Samples were separated by SDS-PAGE blotted on nitrocellulose paper and probed with anti-Flag antibodies. The molecular weight of the protein ladder is shown in the left. Protein bands at the expected size of the fusions are shown in red boxes (lanes 3 to 18)

To evaluate whether SHuffle expressed PDI-GPx7 fusions with a C-terminal Flag tag were soluble and presumably correctly folded, a western blot analysis using anti-Flag tag antibodies was conducted on SHuffle cell lysates expressing various PDI-GPx7 fusions as described in materials and methods. In summary, cells were grown at 30 °C to mid-log, induced with 500 μM IPTG and grown for an additional 5 h. Cells were collected, subjected to lysis by sonication and an aliquot was removed to represent the total lysate (T). The insoluble fraction was pelleted by centrifugation and the supernatant fraction was collected to represent the soluble fraction (S). Samples were boiled in loading buffer, separated by SDS-PAGE and subjected to western blot analysis using anti-Flag tag antibodies (Fig. 3).

As a negative control for cross reactivity of the Flag-tag antibody to proteins within the SHuffle lysate, cells harboring an empty pACYC-Duet vector were used (MB4638). Little to no background of the anti-Flag antibody was observed, indicating that the bands observed in flag-tagged samples are specific to the Flag epitope (Fig. 3, lane 1). SHuffle cells expressing pBAD34-KatG-flag (MB2797) were used as a positive control for the western blot and the major band at the expected size of 83 kDa was observed, indicating that the western blot analysis functioned as expected with little to no cross reactivity (Fig. 3, lane 2). SHuffle cells expressing the various PDI-GPx7 fusion proteins were similarly treated and the resulting blot analysis detected equal amounts of protein in the total and soluble fractions, suggesting PDI-GPx7 fusion proteins are mostly soluble (Fig. 3, lanes 3 to 18, red boxes). Only the PDI-GPx7 fusion cloned from the pear plant resulted in comparatively weak expression, albeit was also soluble (Fig. 3, lanes 15, 16).

Even though the fusions were soluble, significant levels of other species were also observed. The smaller weight products may represent degradation products while the larger species may be mis-oxidized species. This is mostly likely due to the artificial fusions and incorrect folding of eukaryotic proteins in *E. coli*. Yet the majority of the species detected was in the expected size.

Digital analysis of the intensity of the Flag-tag detected protein bands using ImageJ, indicated that the PDI-GPx7 fusions at their expected sizes were on average ~ 50% of the total protein bands in the soluble lysate. Expression of Mollusk and Sponge PDI-GPx7 fusions resulted in predominant protein bands below 30 kDa (Fig. 3, lanes 9–12). These could correspond to cleaved fusion products, resulting in GPx7 (~ 20 kDa) and PDI (~ 55 to 61 kDa).

### Redox state analysis of PDI-GPx7 fusion pairs

Both PDI and GPx7 are redox-active enzymes involved in the formation of disulfide bonds. In their native ER compartment, the active site cysteines of mammalian (Appenzeller-Herzog and Ellgaard 2008) or yeast (Vitu et al. 2010) PDI within the CXXC motif are maintained mostly in their oxidized, disulfide-bonded state. PDI is maintained in its oxidized state by Ero1 (Ramming et al. 2015), while GPx7 is oxidized by H_2_O_2_, which in turn oxidizes PDI (Wang et al. 2014). In this study, an artificial redox pathway was engineered within SHuffle cells to promote disulfide bond formation relay via H_2_O_2_ → GPx7 → PDI → Humira. In order to couple the oxidation of GPx7 to the oxidation of PDI, the protein pair was expressed as a fusion.

**Fig. 4:**
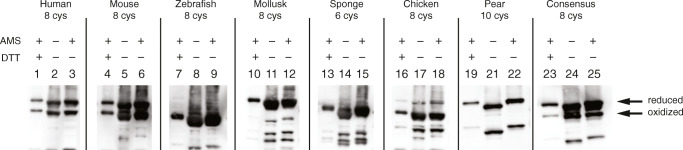
Redox states of SHuffle expressed Gpx7-PDI fusions. SHuffle cells expressing various PDI-GPx7 fusions were precipitated with TCA and pellets were either resuspended in loading buffer representing the fully oxidized species (lanes 2, 5, 8, 11, 14, 17, 21, 24) or samples were first reduced with DTT followed by AMS alkylation representing the fully reduced species (lanes 1, 4, 7, 10, 13, 16, 19, 23). Redox state of the fusions was evaluated by resuspending the samples in loading buffer with AMS (lanes 3, 6, 9, 12, 15, 18, 22, 25). The number of cysteines in the fusions is shown

PDI-GPx7 fusions contain two CXXC active sites in PDI and one in GPx7, totaling six cysteines involved in redox reactions. Only the fusion from sponge (*Amphimedon queenslandica*) has only redox-active cysteines, while all other fusions have 2–4 other presumably structural cysteines. In their native ER compartments, the active site cysteines are mostly in their oxidized, disulfide-bonded state. To investigate the redox states of the cysteines of the PDI-GPx7 fusion, AMS alkylation on soluble cell extracts was performed. AMS alkylates free thiol groups, covalently adding 500 Da per cysteine, resulting in a mobility shift in SDS-PAGE analysis (Berkmen 2012). Western blot analysis of the fusions using anti-Flag tag antibodies revealed that the fusions are mostly in their oxidized state (Fig. 4, lanes 3, 6, 9, 12, 15, 18, 25). Only the poorly expressing PDI-GPx7 fusion from pear (*Pyrus x bretschneideri*) was detected to be mostly in its reduced state (Fig. 4, lane 22). A small shift in mobility is observed with the fusions when cell extracts were treated with AMS, indicating that the majority of the cysteines in the fusions are oxidized. When the mobility of AMS treated samples are compared to reduced samples treated with DTT (Fig. 4, lanes 1, 4, 7, 10, 13, 16, 23) a significant difference in mobility is observed. Supporting the conclusion that except for the fusion from pear, the rest of the PDI-GPx7 fusions are expressed mostly in their active, oxidized state in the cytoplasm of SHuffle cells.

### Selection of PDI-GPx7 fusion to fold Humira, in a shake-flask expression condition

**Fig. 5:**
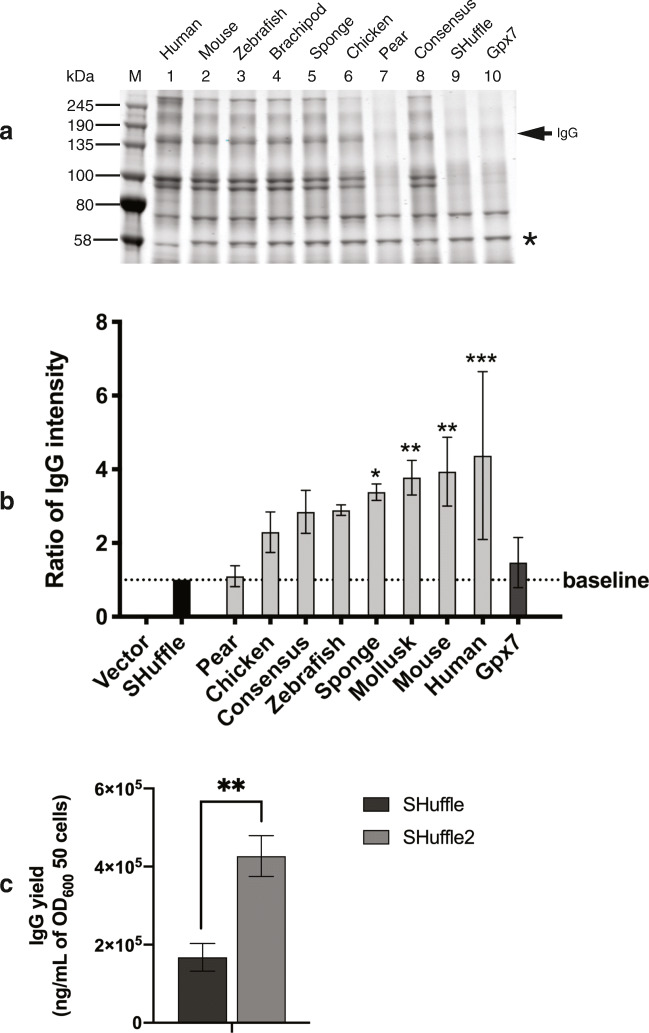
SHuffle expression of Humira IgG is improved by Gpx7-PDI fusions. **a** Effects of PDI-GPx7 fusions on the folding of Humira IgG is evaluated by protein A purification from soluble SHuffle lysates grown in shake-flask conditions. Samples were separated in non-reducing SDS-PAGE. The expected size of Humira IgG (150 kDa) is indicated with an arrow. Data are representative of three independent experiments. **b** Evaluation of PDI-GPx7 fusions on the Humira IgG folding conducted by quantifying the intensity of the band representing Humira to a contaminating band used to normalize loading amounts (*), (*n* = 3). One-way ANOVA with alpha = 0.05, *** *p* value ≤ 0.001, ** *p* value ≤ 0.01, * *p* value ≤ 0.05. **c** Yields of protein A purified Humira antibody, produced in SHuffle or SHuffle2 strains grown in shake flasks from three independent cultures (*n* = 3). Unpaired *t* test with alpha = 0.05, ** *p* value ≤ 0.01

To select for the optimal PDI-GPx7 fusion which has the highest impact on the folding of Humira IgG, SHuffle strains expressing pHumira and pET-Duet plasmids expressing the various PDI-GPx7 fusions were grown, induced and Humira IgG was purified from cell extracts as described in materials and methods. Protein A purified extracts normalized to cell density were separated by molecular weight in non-reducing SDS-PAGE (Fig. 5a). Compared to SHuffle cells expressing Humira with an empty vector (Fig. 5a, lane 9, MB6209), co-expression of PDI-GPx7 fusions dramatically increased the protein band around the expected size of Humira IgG at ~ 150 kDa (Fig. 5a, lanes 1–8). This process was repeated in three independent biological replicates. Intensity of the protein band representing the full-length IgG at ~ 150 kDa was standardized to a contaminating band found in all samples at ~ 58 kDa (Fig. 5a, protein band marked by a star (*)). The average intensity of Humira IgG from purified samples was calculated and presented in order of improvement (Fig. 5b). To address whether GPx7 is directly involved in the folding of Humira IgG independently of PDI, the MB6210 strain expressing GPx7 on its own was also evaluated for Humira IgG expression. Unlike the expression of the PDI-GPx7 fusion, the expression of Gpx7 alone had marginal improvement on the folding of Humira IgG (Fig. 5b). To confirm the positive impact of the human PDI-GPx7 on the high level of Humira IgG production, ELISA assays were performed towards the antigen (TNFα), from purified IgG extracts produced in SHuffle wt cells (MB6209) or in the SHuffle2 strain co-expressing human PDI-GPx7 fusion (MB6201). Protein concentration of the purified fraction was measured for each extract and the final yields of IgG obtained in the purified fraction were calculated as 427 mg/L Humira from SHuffle2 (MB6201) and 168 mg/L Humira for SHuffle (MB6209), an improvement of more than 2-fold in SHuffle2 grown in shake flask (Fig. 5c). Taken together, the human PDI-GPx7 fusion demonstrated the highest potential to improve the folding of Humira IgG and was selected for further characterization.

### Evaluation of SHuffle2 in high-density expression of Humira

Production of proteins at large scales is usually conducted in high-density fermenters. It is therefore important to validate the positive impact of human PDI-GPx7 on the folding of Humira IgG, in conditions similar to industrial processes.

**Fig. 6:**
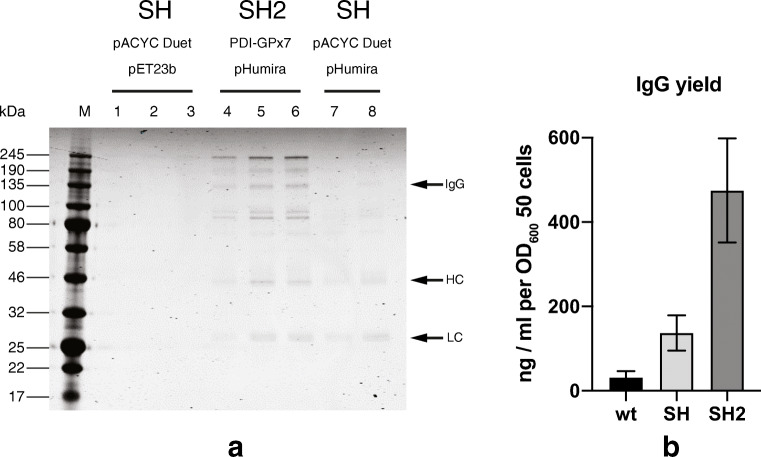
High-density fermentation of Humira IgG expression is improved in SHuffle2. **a** Expression of Humira IgG was evaluated in SHuffle cells harboring empty vector (MB4135 lanes 1–3), in SHuffle cells expressing Humira IgG (MB6209, lanes 7–8), or in SHuffle2 cells expressing Humira IgG and co-expressing human PDI-Gpx7 fusion (MB6201, lanes 4–6). Purified Humira IgG was analyzed in SDS-PAGE under non-denaturing conditions. Full length correctly assembled IgG and unassembled Heavy Chain (HC) and Light Chain (LC) are indicated by arrows. **b** Humira IgG yields from wild type SHuffle cells (wt, MB4135), SHuffle control cells carrying the pACYC Duet plasmid (SH, MB6209), and SHuffle2 cells (SH2, MB6201) grown in high-density from two or three independent cultures

Cells were grown to high density in a Dasgip BioBlock fermenters containing 1 L of complex medium. Three different *E. coli* cells were used to evaluate Humira IgG expression, SHuffle cells with their oxidizing cytoplasm harboring empty vectors as a control for background (MB6209), SHuffle cells expressing Humira IgG (MB4135), and SHuffle2 cells co-expressing human PDI-GPx7 (MB6201). Cells were collected and lysed and the Humira IgG was purified from the soluble lysate of cells, using protein A as described in “Materials and methods.” Humira IgG produced in high density was first analyzed by running an aliquot of purified samples in SDS-PAGE under non-reducing conditions. This process was repeated in triplicates for wild type (wt) and SHuffle2 (SH2) cells and in duplicates for SHuffle (SH) cells (Fig. 6a). The Humira full length correctly assembled IgG tetramer was observed at the expected size of ~ 150 kDa, but as was not the major species. Unassembled light chain (LC, 25 kDa) and heavy chain (HC, 50 kDa) peptides were also observed, indicative that the expression levels are not limiting. Supporting the notion that the limiting factor is the correct assembly of LC and HC, higher molecular weights protein bands above 200 kDa were observed. Previous studies on expression and purification of IgG from SHuffle cells have observed similar mis-assembled IgG species that are mis-oxidized, as these high-molecular bands collapse into LC or HC, under reducing conditions (Leith et al. 2019; Lobstein et al. 2012; Reddy et al. 2018). Binding capacity to Humira IgG’s cognate antigen TNFα was measured by ELISA and IgG concentration of the purified extracts were calculated (Fig. 6b). Results indicated that a 3–4-fold improvement in the final yields of IgG was observed when Humira IgG was expressed in SHuffle 2 compared to parental SHuffle strain. Taking the complete data set presented, the co-expression of the human PDI-GPx7 fusion in SHuffle cells, improve the expression and folding of Humira IgG in an industrially relevant manner.

## Discussion

Expression of antibodies in *E. coli* merges the potentials of antibody-based technologies with *E. coli* genetics. A major limitation in expressing recombinant proteins in the cytoplasm of *E. coli* is the lack post-translational modification systems, such as disulfide bond formation. This study succeeded in coupling metabolic oxidant H_2_O_2_ to disulfide bond formation, via the expression of a synthetic eukaryotic pathway in a genetically engineering prokaryotic host. Success of this synthetic system was demonstrated by improving the yield of the most profitable protein, an IgG against rheumatoid arthritis, Humira.

This preliminary study indicates that the concept of utilizing the oxidation power of H_2_O_2_ to couple to disulfide bond formation, by redox engineering of a eukaryotic pathway into a prokaryotic system is feasible. In order to select the correct eukaryotic redox system, several attempts were made to evaluate the functionality of various eukaryotic systems. A close homolog of GPx7 is GPx8, which shares the highest structural similarity and has similar capacity to oxidize PDI via H_2_O_2_ but has different biological roles in stress response. This study focused on GPx7 as it has one order of magnitude higher reactivity than GPx8 towards H_2_O_2_ and is not anchored to the membrane as GPx8 is predicted to have a type I transmembrane domain (Wang et al. 2014). Another class of peroxidase that can oxidize PDI is Prx4 (Tavender et al. 2010). Prx4 can also catalyze de novo disulfide bond formation by reducing H_2_O_2_ but it expresses poorly in SHuffle cells (data not shown) and unlike GPx7, has a minor phenotype in knock-out mouse (Iuchi et al. 2009). Further, oxidation of a substrate protein by PDI was significantly slower in the presence of Prx4, compared to GPx7 (Nguyen et al. 2011). These preliminary attempts resulted in picking PDI-GPx7 fusions as the candidates to engineer a new SHuffle system.

Even though elevated levels of H_2_O_2_ were confirmed in the cytoplasm of SHuffle (Reuter et al. 2019), the exact concentration of this transient and highly reactive oxidant is not known. It may therefore be possible that the levels of H_2_O_2_ are not sufficient and increasing the availability of H_2_O_2_ can improve GPx7 driven disulfide bond formation. An enzymatic production of H_2_O_2_ was attempted by expressing ER resident Ero1 in pET-Duet to increase cytoplasmic concentration of H_2_O_2_. Ero1 can consume O_2_ to produce H_2_O_2_, but preliminary evidence using human Ero1 in SHuffle2 did not increase levels of Humira production (data not shown). Similarly, exogenous addition of H_2_O_2_ did not result in any observable improvement in Humira IgG folding (data not shown). This may be due to the highly reactive nature of H_2_O_2_ and thus perhaps a triple fusion of PDI-GPx7-Ero1 may supply H_2_O_2_ to GPx7 in close proximity. Further improvements in the production of Humira IgG could be also achieved by expressing a mutant version of Humira with a single silent mutation (Val_216_ GTG mutated to synonymous GTT), as this version does not result in the re-initiation of the heavy chain (Leith et al. 2019).

Synthetic pathways often are trial and error attempts at engineering an artificial gene circuit and are not optimized by the selective processes of evolution. Although the designs of these systems can be guided by bioinformatic and system biology, functionality of these artificial systems often are specific for certain substrates under certain conditions. This was the case for the artificial PDI-GPx7 coupled redox pathway, engineered to improve IgG folding in SHuffle cells. Although great success was achieved in improving the folding of Humira IgG, little to no improvement was observed for two other IgG molecules tested, NIST mAb (Reddy et al. 2018) and anti-MBP (Lobstein et al. 2012) (data not shown). Many other IgG molecules and derivatives need to be evaluated under various expression conditions, to discern the specificity of the PDI-Gpx7 coupled disulfide bond formation in SHuffle cells.

## Electronic supplementary material

ESM 1 (PDF 477 kb)
