# Supplementary material for: Improved production of Humira antibody in the genetically engineered Escherichia coli SHuffle, by co-expression of human PDI-GPx7 fusions
Source: Appl Microbiol Biotechnol. 2020 Sep 30;104(22):9693–706. doi: 10.1007/s00253-020-10920-5 (PMC7595990; doi:10.1007/s00253-020-10920-5)
Supplement: Supplementary file 1 — (PDF 477 kb) [file 253_2020_10920_MOESM1_ESM.pdf]

Applied Microbiology and Biotechnology

"Improved production of Humira antibody in the genetically engineered *Escherichia coli* SHuffle, by co-expression of human PDI-GPx7 fusions."

Marine Lénon[1,2], Na Ke[1], Cecily Szady[1], Hassan Sakhtah[1], Guoping Ren[1], Bruno Manta[1,3], Bryce Causey[1] and Mehmet Berkmen[1*]

[1] New England Biolabs, 240 County Road, Ipswich, MA 01938, USA

[2] Current address: Department of Microbiology, Stress Adaptation and Metabolism in Enterobacteria Unit, UMR CNRS 2001, Institut Pasteur, 25-28 Rue du Dr Roux, 75015, Paris, France

[3] Current address: Facultad de Medicina, Departamento de Bioquímica and Centro de Investigaciones Biomédicas, Universidad de la República, CP 11800 Montevideo, Uruguay.

*Correspondence: Phone: (978) 380-7519; Fax: (978) 921-1350; Email: berkmen@neb.com

```
Human          ------------------------------------------------------------   0
Mouse          ------------------------------------------------------------   0
Chicken        ----------------------------------------------------------ML   2
Zebrafish      ------------------------------------------------------------   0
Mollusk        ----------------------METSMSPKICPKNILNVVLFSFFFVNIFNEILCGPATI   38
Sponge         ------------------------------------------------------------   0
Pear           MASMSFSATFSTPSQSFSKAKAKISSNSSASCPPMACW-IKFS--FGSS-----------   46
Consensus      ------------------------------------------------------------   0

Human          ---------------------MVAATVAAAWLLLWAAACAQQEQDFYDFKAVNIRGKLVS   39
Mouse          ----------------------MVAAVATAWLLLWAAACAQSEQDFYDFKAVNIRGKLVS   38
Chicken        DPEGSVEHKPSSSFPKVFLIPLAMLLAITALLLLAFSATQQKETDFYTFKVVNIRGKLVS   62
Zebrafish      ---------------------MGMFLR-AFTLIILLCLLEAKQKDFYTFKVVNSRGRLVS   38
Mollusk        DPLK-VKKKPGYRPS------TGGLQPGEIVYEDVTPTTDEPEKDFYMFSVTDIKGKTVS   91
Sponge         -------------MEQVFF--------LSLFCLICGALASSGTETFYSLSAKDITGKMIG   39
Pear           ---KSALFRNGLSLQSENF--NGVLIKSR---SFGVYARAATERSLYDFTVKDIDGKDVP   98
Consensus      ----------------------------------------QQEQDFYDFKAVNIRGKLVS   20

                         ↓                                   ↓
Human          LEKYRGSVSLVVNVASECGFTDQHYRALQQLQRDLGPH-HFNVLAFPCNQFGQQEPDSNK   98
Mouse          LEKYRGSVSLVVNVASECGFTDQNYRALQQLQRDLGPH-HFNVLAFPCNQFGQQEPDTNR   97
Chicken        LEKYRGSVSLVVNVASECGFTDSHYKALQQLQKDLGPY-HFNVLAFPCNQFGQQEPDTNK   121
Zebrafish      LEKYRGSVSLAVNVASECGYTDEHYKDLQQLQKDFGPF-HFNVLAFPCNQFGQQEPGSDK   97
Mollusk        LEEYRGMVTLVVNVASECGYTDSHYKALVKLQNTLAPSGKFTVLAFPCNQFGAQEPKDEP   151
Sponge         FERYSGKIVLVVNVASECGYTDYNYIQLNQLQLRYGEE-SLAILAFPCNQFGAQEPAKDS   98
Pear           LSKFKGKVLLVVNVASRCGLTSSNYSELSHLYEKYKPQ-GFEILAFPCNQFGGQEPGSNG   157
Consensus      LEKYRGSVSLVVNVASECGFTDQHYRALQQLQRDLGPH-HFNVLAFPCNQFGQQEPDSNR   79

                                                     ↓
Human          EIESFARRTYSVSFPMFSKIAVTGTGAHPAFKYLAQ-TS---GKEPTWNFWKYLVAPDGK   154
Mouse          EIENFARRTYSVSFPMFSKIAVTGTGAHPAFKYLTQ-TS---GKEPTWNFWKYLVDPDGK   153
Chicken        EIESFARKTYGASFPMFSKVAVSGAGAIPAFKYLID-ST---GEEPTWNFWKYLVDPNGK   177
Zebrafish      EIDSFVRRVYGVSFPIFSKIAVVGIGANNAYKYLVE-AS---RKEPTWNFWKYLIDTDGK   153
Mollusk        SIEKFAKEKYGVNFPMFSKINVVEKDIPEAWKFLED-FS---RLVPNWNFWKYLINPSGH   207
Sponge         EINSMIRYKYKPQFPLFSKVNVTGESQSEVYRFLINSTN---GLEPKWNFCKYLLDRDGL   155
Pear           EIKQFACTRFKAEFPIFDKVDVNGPSTAPVYQFLKSSAGGFLGDIIKWNFEKFLVDKNGK   217
Consensus      EIESFARRTYSVSFPMFSKVAVTGTGAHPAFKYLTE-TS---GKEPTWNFWKYLVAPDGK   135

Human          VVGAWDPTVSVEEVRPQITALVRKLILL------KREDL   187
Mouse          VVGAWDPTVPVAEIKPRITEQVMKLILR------KREDL   186
Chicken        VVKAWDSTVSVEEIRPHVTELVRKIILK------KKDEL   210
Zebrafish      VVDAWGPEVSVKEIRPRITEMVRKLIIK------RKEEL   186
Mollusk        VIATWGPWIPVEDVIEQITEAVHAAADDLVVSDTIHEEL   246
Sponge         VVQFFDQGKTFDKIYDSIDGLLYRKR----------REL   184
Pear           VVERYPPTTSPFQIEKDIQKLVA----------------   240
Consensus      VVGAWDPTVSVEEIRPQITALVRKLILK------KREDL   168
```

Figure S1. Sequence alignment of the candidate GPx7 proteins. The alignment was obtained using ClustalW and manually edited. Amino acids whose identify threshold is above 80% are shaded in gray. Cysteines are highlighted in yellow, signal peptide is underlined in red, dimer interface is underlined in black, arrows point at catalytic residues determined from Conserved Domain Database (https://www.ncbi.nlm.nih.gov/Structure/cdd/cdd.shtml) and the classical C-terminal ER retention signal sequence KDEL for lumen bound proteins is underlined in green.

Table S1. Primers used in construction of plasmids.

Name	Sequence (5' to 3')
NK1012-flag-r	caagcttgGTCGACTTActtgtcatcgtcatccttgtaatcgatgtcatg
NK1013-c-f	GAAAAAACGTGAAGACCTGgactacaaagaccatgacggtgattataaagat
NK1014-pxb-f	CTGGTTGCGGCGgactacaaagaccatgacggtgattataaagat
NK1015-aqu-f	GCGCCGTGAACTGgactacaaagaccatgacggtgattataaagat
NK1016-lak-f	CATTCACGAGGAGCTGgactacaaagaccatgacggtgattataaagat
NK1017-dre-f	CGCAAGGAAGAGCTGgactacaaagaccatgacggtgattataaagat
NK1018-gga-f	CCTGAAGAAGAAAGATGAACTGgactacaaagaccatgacggtgattataaagat
NK1019-mmu-f	GTAAGCGTGAAGACCTGgactacaaagaccatgacggtgattataaagat
NK1020-has-f	GAAACGTGAGGACCTGgactacaaagaccatgacggtgattataaagat
5'-nsGpx7 (NcoI)	ggtCCATGGccCAGCAGGAGCAGGACTTC
3'-nsGpx7 (HindIII)	ggtAAGCTTATAAGTCTTCTCGCTTCAGTAG

Table S2. Construction of plasmids used in this study.

Plasmid	Template	Primer pair	Cloning method
pACYC Duet - PDI-hsa Gpx7 - Flag	pACYC Duet - PDI-hsa Gpx7*	NK1012-FLAG-r<br>NK1020-has-f	NEBuilder® HIFI DNA (NEB, cat. No. E5520)
pACYC Duet - PDI-mmu Gpx7 - Flag	pACYC Duet - PDI-mmu Gpx7*	NK1012-FLAG-r<br>NK1019-mmu-f	
pACYC Duet - PDI-lak Gpx7 - Flag	pACYC Duet - PDI-lak Gpx7*	NK1012-FLAG-r<br>NK1016-lak-f	
pACYC Duet - PDI-aqu Gpx7 - Flag	pACYC Duet - PDI-aqu Gpx7*	NK1012-FLAG-r<br>NK1015-aqu-f	
pACYC Duet - PDI-dre Gpx7 - Flag	pACYC Duet - PDI-dre Gpx7*	NK1012-FLAG-r<br>NK1017-dre-f	
pACYC Duet - cPDI-Gpx7 - Flag	pACYC Duet - cPDI-Gpx7*	NK1012-FLAG-r<br>NK1013-c-f	
pACYC Duet - PDI-gga Gpx7 - Flag	pACYC Duet - PDI-gga Gpx7*	NK1012-FLAG-r<br>NK1018-gga-f	
pACYC Duet - PDI-pxb Gpx7 - Flag	pACYC Duet - PDI-pxb Gpx7*	NK1012-FLAG-r<br>NK1014-pxb-f	
